# Iron is a specific cofactor for distinct oxidation- and aggregation-dependent Aβ toxicity mechanisms in a *Drosophila* model

**DOI:** 10.1242/dmm.019042

**Published:** 2015-07-01

**Authors:** Stanislav Ott, Nikolas Dziadulewicz, Damian C. Crowther

**Affiliations:** 1 Department of Genetics, University of Cambridge, Downing Street, Cambridge CB2 3EH, UK; 2 MedImmune Limited, Aaron Klug Building, Granta Park, Cambridge CB21 6GH, UK

**Keywords:** Iron, Metal, Amyloid beta peptide, Alzheimer's disease, Oxidative stress, *Drosophila*

## Abstract

Metals, including iron, are present at high concentrations in amyloid plaques in individuals with Alzheimer's disease, where they are also thought to be cofactors in generating oxidative stress and modulating amyloid formation. In this study, we present data from several *Drosophila* models of neurodegenerative proteinopathies indicating that the interaction between iron and amyloid beta peptide (Aβ) is specific and is not seen for other aggregation-prone polypeptides. The interaction with iron is likely to be important in the dimerisation of Aβ and is mediated by three N-terminal histidines. Transgenic fly lines systematically expressing all combinations of His>Ala substitutions in Aβ were generated and used to study the pathological role of these residues. Developmental eye phenotypes, longevity and histological examinations indicate that the N-terminal histidines have distinct position-dependent and -independent mechanisms. The former mediate the toxic effects of metals and Aβ aggregation under non-oxidising conditions and the latter are relevant under oxidising conditions. Understanding how Aβ mediates neurotoxic effects *in vivo* will help to better target pathological pathways using aggregation blockers and metal-modifying agents.

## Abstract (precis)

**Highlighted Article:** Iron plays a specific role in amyloid beta peptide (Aβ) toxicity in a *Drosophila* model system. Aβ and iron exhibit distinct toxicity mechanisms in oxidising and non-oxidising environments.

## INTRODUCTION

The primary role of amyloid beta peptide (Aβ) in the aetiology of Alzheimer's disease (AD) is supported by a wealth of genetic and biochemical data (Hardy and Selkoe, 2002; Walsh et al., 2002): not only is familial AD known to be caused by mutations that favour aggregation-prone isoforms of Aβ (Goate et al., 1991; Levy-Lahad et al., 1995; Li et al., 1995; Rogaev et al., 1995; Sherrington et al., 1995; Suzuki et al., 1994) but also, conversely, disease risk is reduced by a mutation that slows peptide generation (Jonsson et al., 2012). Such observations have prompted many groups to investigate how Aβ mediates its toxic effects in AD. Despite significant advances in the field, the pathogenic mechanism is incompletely understood and is likely to be complex.

Aβ is a member of a family of polypeptides that are prone to disease-associated amyloid formation. Other members of this family include tau, which is involved in tauopathies and AD (Spillantini and Goedert, 2013), polyQ peptides, which underpin Huntington's disease (HD) (Imarisio et al., 2008), and TDP43, which is linked to motor neurone disease (Ling et al., 2013). There is accumulating evidence that Aβ, along with these amyloid-forming polypeptides, can exert a generic toxicity on cells (Baglioni et al., 2006). The toxicity is generic in two regards: first, oligomeric aggregates of peptides damage a wide variety of mammalian cell types (Barucker et al., 2014; Jang et al., 2014; Kinghorn et al., 2006; Kruppa et al., 2013); second, oligomers comprising a wide range of polypeptides can exhibit cytotoxicity (Bucciantini et al., 2002; Cascella et al., 2013; Demuro et al., 2005; Newington et al., 2011; Zhao et al., 2009). Studies into the toxicity of amyloid aggregates have been undertaken in a wide range of model systems, not only in mammalian tissues and cells but also using vertebrate and invertebrate *in vivo* model systems. In our *Drosophila* model of Aβ toxicity we have demonstrated a robust correlation between the propensity of an Aβ peptide variant to form oligomeric aggregates and its *in vivo* toxicity, as estimated by longevity and locomotor phenotypes (Luheshi et al., 2007). The mechanism of oligomer toxicity might be mediated by particular biophysical characteristics; indeed, there is evidence that amyloid oligomers share structural features that are linked to their toxic mechanism (Kayed et al., 2007). One proposed mechanism is that oligomeric aggregates are able to degrade the barrier properties of membranes, resulting in calcium fluxes (Demuro et al., 2005; Yoshiike et al., 2007). By contrast, there is also evidence for pathogenic mechanisms involving binding to specific cell surface receptors – an example being the prion protein (Barry et al., 2011; Kudo et al., 2011; Lauren et al., 2009; Younan et al., 2013).

However, there are many more aspects to Aβ toxicity and of particular interest is the pathogenic role of redox-active metal cofactors in generating oxidative stress in the brain. Our work and that of others indicates that the accumulation of Aβ in the brain correlates with oxidative damage to proteins, lipids and DNA from the earliest stages of the disease process (Butterfield et al., 2001; Markesbery et al., 2005). Interest has focussed on the role of Aβ aggregates as redox-active centres that are able to complex with metal ions such as copper (Du et al., 2014; Huang et al., 1999b; Jiang et al., 2007) and iron (Everett et al., 2014; Huang et al., 1999a). The affinity of Aβ for metal ions is thought to be mediated in large part by imidazole nitrogens of the three N-terminal histidine residues (Nair et al., 2010; Viles, 2012). Indeed, potential ligands such as copper and iron approach millimolar concentrations within amyloid plaques (Lovell et al., 1998) in AD, leading to clinical trials of metal chelators as potential therapies (Ritchie et al., 2003). Although the clinical efficacy of metal chelators, such as clioquinol, needs further investigation, their benefits in disease models have been documented in both mammalian (Cherny et al., 2001) and invertebrate (Rival et al., 2009) systems. The origin of the bulk of the oxidative stress in AD is unknown, but there are a number of potential sources: first, it may be generated by the redox-active Aβ aggregates themselves (Huang et al., 1999a,b; Jiang et al., 2007); second, as a product of disease-linked mitochondrial dysfunction (Hirai et al., 2001; de la Monte and Wands, 2006; Onyango et al., 2006; Zempel et al., 2010); or third, as a result of an inflammatory response to amyloid deposition (Perry et al., 2010).

**TRANSLATIONAL IMPACT**

**Clinical issue**

The prevalence of Alzheimer's disease (AD) is increasing and yet there are no disease-modifying therapies. Much evidence supports a role for amyloid beta peptide (Aβ) in the disease process; however, too little is known about its neurotoxic mechanism. Metals such as iron are found at high levels in Aβ plaques in patients’ brains and are thought to mediate the oxidative stress and other toxic processes that accompany the peptide's production and aggregation. Understanding how metals mediate toxicity in AD is important to better elucidate AD pathophysiology and to develop effective therapies.

**Results**

In this study, the authors used different *Drosophila* models of neurodegenerative diseases and found that the interaction of iron with aggregation-prone polypeptides is AD-specific (i.e. for Aβ) and is not seen in models of frontotemporal dementia, Huntington's disease and motor neurone disease. The interaction of iron with Aβ appears to occur at an early stage in the aggregation process, possibly during the formation of the peptide dimer. There are three histidines in Aβ that are thought to mediate the effects of metals. When these histidines are systematically substituted with alanines it is possible to dissect their individual contribution to the overall toxicity. The contribution of each histidine to toxicity is different in oxidising versus non-oxidising environments.

**Implications and future directions**

This study suggests that *in vivo* sensitivity to oxidative stress is determined simply by the total number of histidines in the Aβ peptide, irrespective of their position. This predicts that a reduction in total Aβ in the brain will most effectively reduce oxidative damage in AD. By contrast, in non-oxidising conditions, toxic Aβ deposition and the role of metals in generating toxicity depend on the histidines' positions in the peptide. These findings are likely to indicate a role for peptide conformation in determining toxicity. Specific toxic conformations could be targeted therapeutically using small molecules and biological therapeutics such as antibodies.

Our previous studies have shown that an increase in the chelation of iron is able to prolong the life of flies that express aggregation-prone Aβ isoforms. Moreover, the expression of aggregation-prone Aβ also leads to higher levels of oxidative damage in the flies (Rival et al., 2009). The production of peroxide and superoxide, and their conversion to the even more potent hydroxyl radical by iron-mediated Fenton chemistry, is thought to be crucial in amplifying oxidative damage (Khan et al., 2006; Molina-Holgado et al., 2007; Rival et al., 2009; Smith et al., 2000; Zecca et al., 2004). In addition, metals such as copper and iron have been shown to modify the amyloid aggregation process, suggesting that the effects of metal binding to Aβ are not limited to redox chemistry alone. *In vitro* studies have shown that at low pH metals favour amyloid fibres, whereas higher pH favours pre-amyloid intermediates (Atwood et al., 1998; Jiao and Yang, 2007; Liu et al., 2011; Miura et al., 2000; Zou et al., 2001). The concentration of metals may also determine the type of aggregates that are preferentially formed (Viles, 2012). In this paradigm, metals enhance toxicity as the highly toxic oligomeric aggregates are stabilised.

Here, we use our *Drosophila* model of Aβ toxicity to investigate the contributions of these various pathogenic mechanisms *in vivo*. We assess the extent to which the metal enhancement of toxicity, as seen with Aβ, can be generalised to other aggregation-prone polypeptides. We then systematically assess the role of the histidine residues in Aβ and how they mediate distinct metal-mediated, oxidative and protein aggregation stresses.

## RESULTS

### The iron-binding protein ferritin is a specific suppressor of Aβ toxicity *in vivo*

We have previously shown that the expression of ferritin subunits and treatment with iron chelators are able to rescue Aβ-induced toxicity in our *Drosophila* model system (Rival et al., 2009). Here, we studied whether the benefits of iron chelation are also apparent in *Drosophila* models of other prominent neurodegenerative disorders. We tested whether ferritin is able to suppress the rough-eye phenotypes of flies that express: (1) the dementia-linked variant of tau (tau R406W) (Wittmann et al., 2001); (2) an expanded polyglutamine polypeptide (Q48), as a model of HD; and (3) TAR DNA-binding protein 43 (TDP43), which is implicated in neurodegenerative disorders including AD and motor neurone disease (Gendron et al., 2013).

**Fig. 1.**
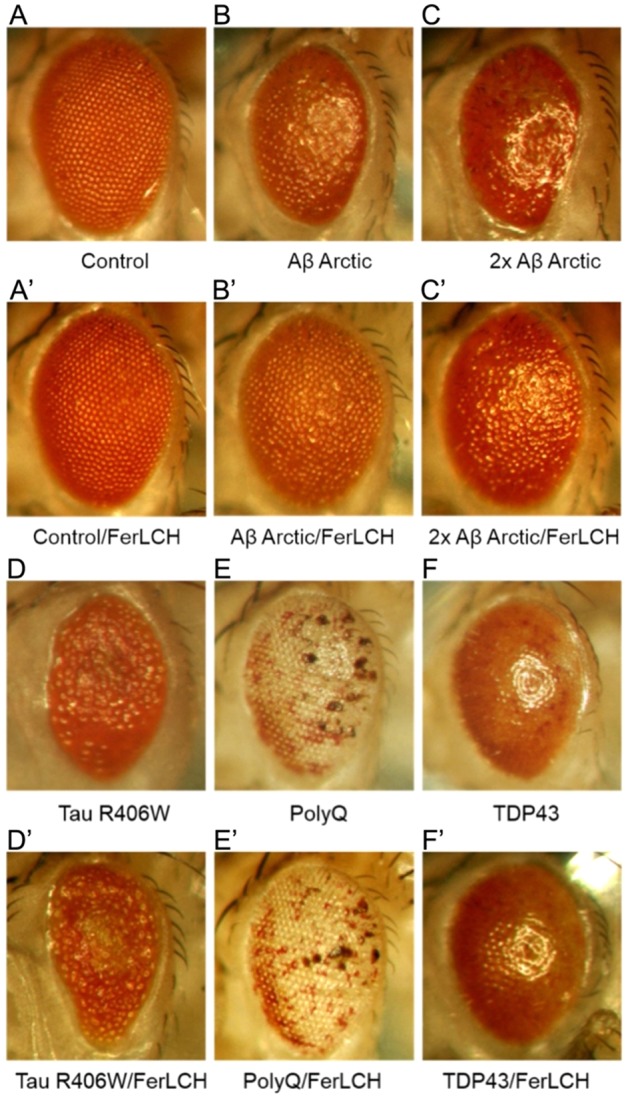
**Ferritin light chain rescues the rough-eye phenotype caused by Aβ expression but has no effect on the equivalent phenotypes caused by tau, polyQ or TDP43.** Compared with control flies (A), the expression of one (B) or two (C) transgenes encoding Aβ_42_ Arctic caused a rough-eye phenotype when expressed with GMR-gal4. The expression of FerLCH had no effect on the controls (A′) but significantly reduced the rough-eye phenotype in flies expressing Aβ (B′,C′). By contrast, similar co-expression of FerLCH with tau R406W, polyQ or TDP43 did not rescue the rough-eye phenotype (D-F′).

As expected, the co-expression of ferritin light chain (FerLCH) rescued the moderate rough-eye phenotype in flies expressing Aβ_42_ Arctic (Fig. 1B-C′). However, no similar rescue was observed when ferritin was co-expressed with tau R406W, Q48 or TDP43 (Fig. 1D-F′). Expression of ferritin heavy chain (FerHCH) had no effect on the rough-eye phenotype for any genotype (data not shown).

**Fig. 2.**
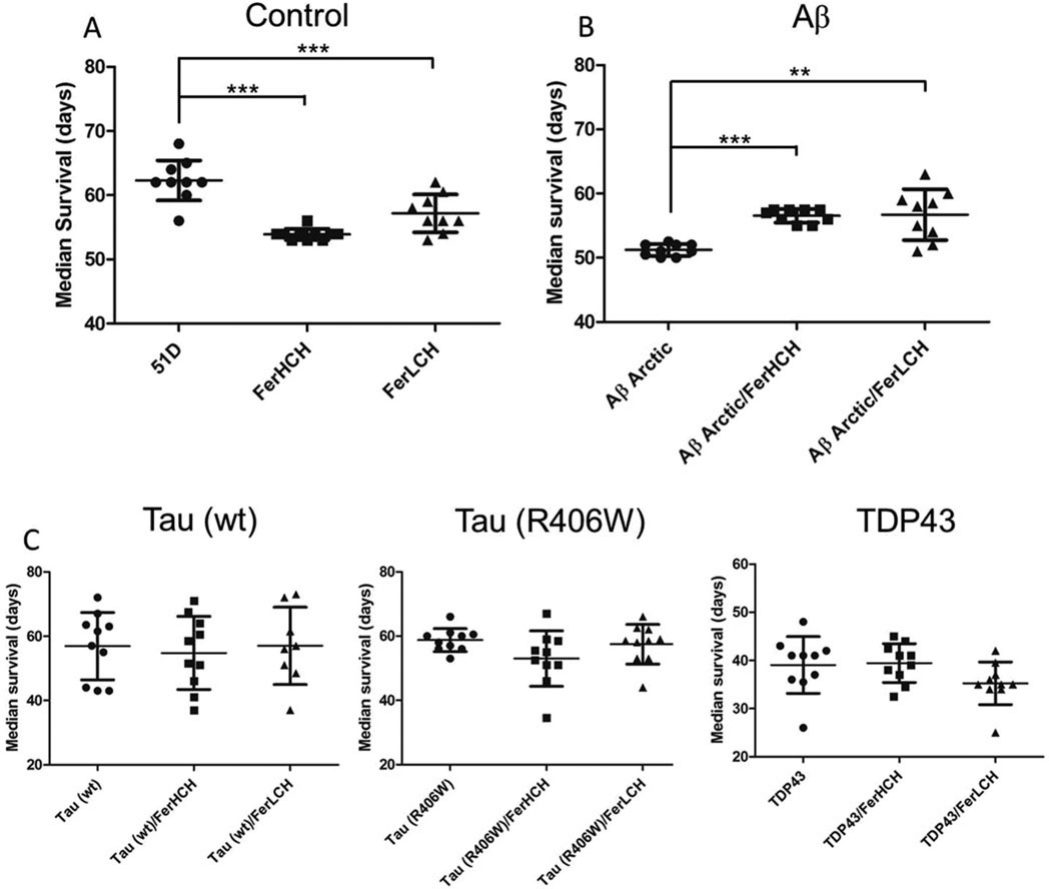
**Ferritin-mediated longevity rescue is Aβ specific.** (A) Expression of ferritin subunits in control flies (51D) was moderately toxic and reduced longevity. (B) By contrast, the co-expression of ferritin subunits prolonged the lifespan of flies expressing Aβ_42_ Arctic. (C) Ferritin co-expression was unable to rescue the decrease in longevity associated with the expression of wild-type and mutant tau or TDP43. Experiments were performed with at least 90 flies per genotype and repeated twice. Differences in median survivals were tested for significance using the Mann–Whitney test (**P<0.01, ***P<0.001). Error bars show s.d.

To confirm the Aβ-specific nature of the ferritin rescue, we expressed the same aggregation-prone polypeptides with or without ferritin pan-neuronally in the fly brain and monitored their longevity. Concordant with the results of the rough-eye assessments, ferritin prolonged the median lifespan of flies expressing Aβ_42_ Arctic (Fig. 2B); by contrast, this rescue was not observed in flies expressing wild-type tau, R406W tau or TDP43 (Fig. 2C). Additionally, we found that pan-neuronal expression of Q48 was lethal and that this phenotype was not rescued by ferritin. No lifespan extension was observed in control flies expressing ferritin subunits (Fig. 2A); indeed, the median survival of these ferritin-expressing flies was significantly reduced. Taken together, the rough-eye and longevity results indicate that ferritin-mediated rescue is specific to Aβ and that ferritin is not beneficial in control flies or in flies expressing other aggregation-prone proteins.

### Iron modifies Aβ aggregation *in vitro* and *in vivo*

The aggregation mechanism for any polypeptide is likely to begin with the initial formation of a dimer (Chiti and Dobson, 2006). To determine whether iron chelation affects this initial step we compared the effects of iron on the aggregation of partially purified recombinant Aβ versus tandem Aβ. The latter is an artificial polypeptide constructed from two copies of Aβ monomers that are connected together by a 12 amino acid glycine-rich linker. By tethering two Aβ molecules, we increase the local concentration of the peptides and predictably accelerate the formation of dimeric aggregates. Previous work has indicated that tandem Aβ_42_ has an oligomer-rich aggregation mechanism and is particularly toxic, whereas tandem Aβ_40_ does not employ this mechanism and is not toxic (Speretta et al., 2012).

**Fig. 3.**
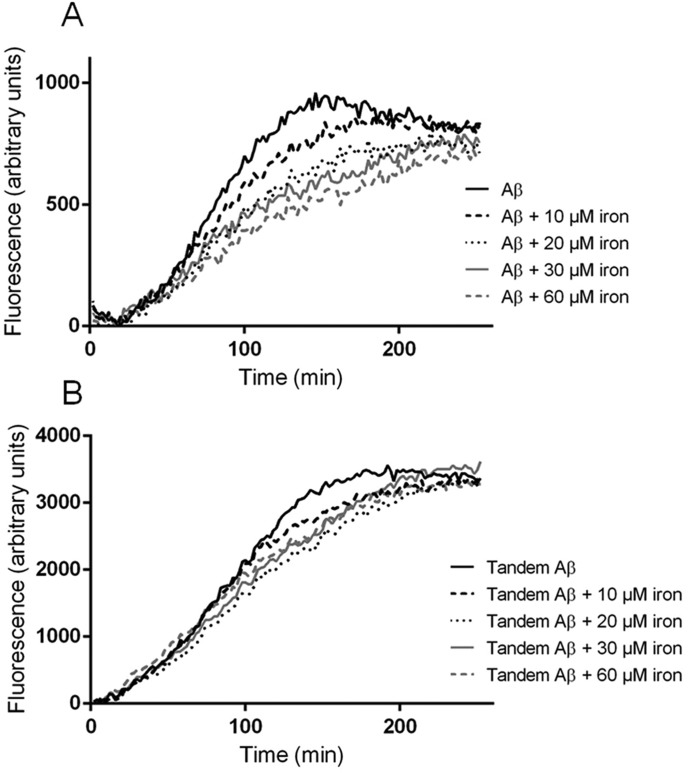
**Amyloid generation by monomeric and tandem Aβ is modulated differently by iron.** (A) In the absence of iron, partially purified recombinant Aβ_42_ generated a ThT fluorescence signal similar to that of synthetic Aβ_42_. Increasing concentrations of iron chloride produced a characteristic delay in the ThT signal. (B) The signal generated by the tandem Aβ_42_ bacterial extract throughout aggregation was not retarded by equivalent concentrations of iron chloride. Values plotted are the mean of three technical replicates. The experiment was performed three times.

**Fig. 4.**
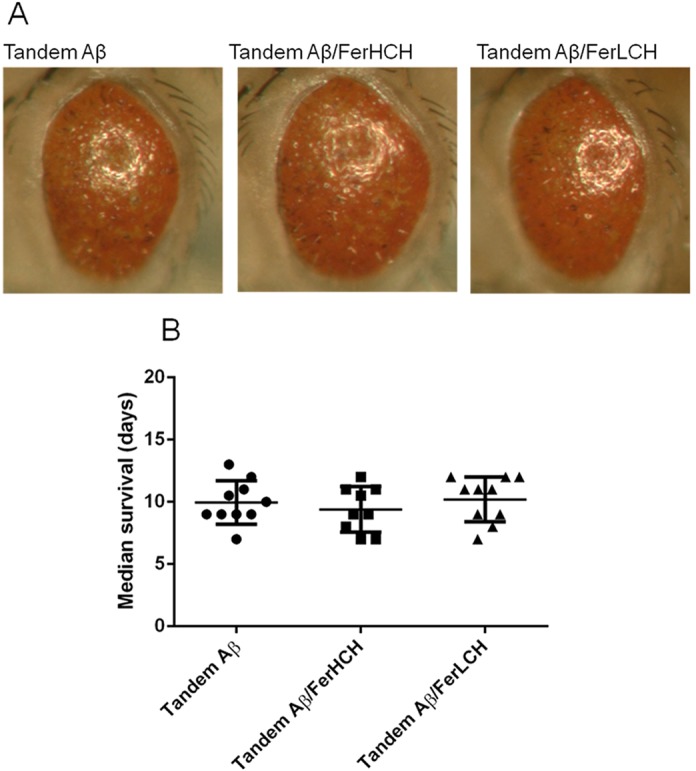
**Co-expression of ferritin subunits does not rescue tandem Aβ_42_ toxicity.** (A) Neither FerLCH nor FerHCH co-expression suppressed the rough-eye phenotype induced by tandem Aβ_42_ driven in the eye by GMR-gal4. Crosses were performed at 25°C and maintained at 29°C until eclosion. (B) In agreement with the rough-eye assessment, pan-neuronal elav^c155^-gal4-driven expression of FerLCH or FerHCH also did not prolong the lifespan of flies co-expressing tandem Aβ_42_. Crosses were performed at 18°C and maintained at 25°C after hatching.

We initially studied the relative effects of iron on Aβ_42_ and tandem Aβ_42_ aggregation using an *in vitro* system. Although tandem Aβ could not be synthesized chemically due to its high aggregation propensity, we were able to express it as inclusion bodies in *E. coli* (supplementary material Figs S1 and S2) (Walsh et al., 2009). By isolating Aβ-containing inclusion bodies and solubilising them in dimethyl sulfoxide (DMSO), we were able to partially purify both peptides. As we previously observed for pure synthetic Aβ_42_ (Liu et al., 2011), the addition of iron to the bacterial extracts of monomeric Aβ progressively delayed thioflavin T (ThT) signal generation, indicating a delay in the formation of mature amyloid structures (Fig. 3A). By contrast, the addition of iron to a solution of tandem Aβ_42_ did not significantly change the kinetics of the ThT signal over a comparable concentration range (Fig. 3B). This relative insensitivity of the tandem Aβ peptide to the presence of iron is concordant with our results obtained with *Drosophila*, in which the toxicity of tandem peptides was not rescued by ferritin co-expression (Fig. 4). These data suggest that iron is able to modify an early, rate-limiting dimerisation step for (monomeric) Aβ (Viles, 2012), an effect that is redundant for tandem Aβ because of the acceleration afforded by the tethering of the two peptides.

**Fig. 5.**
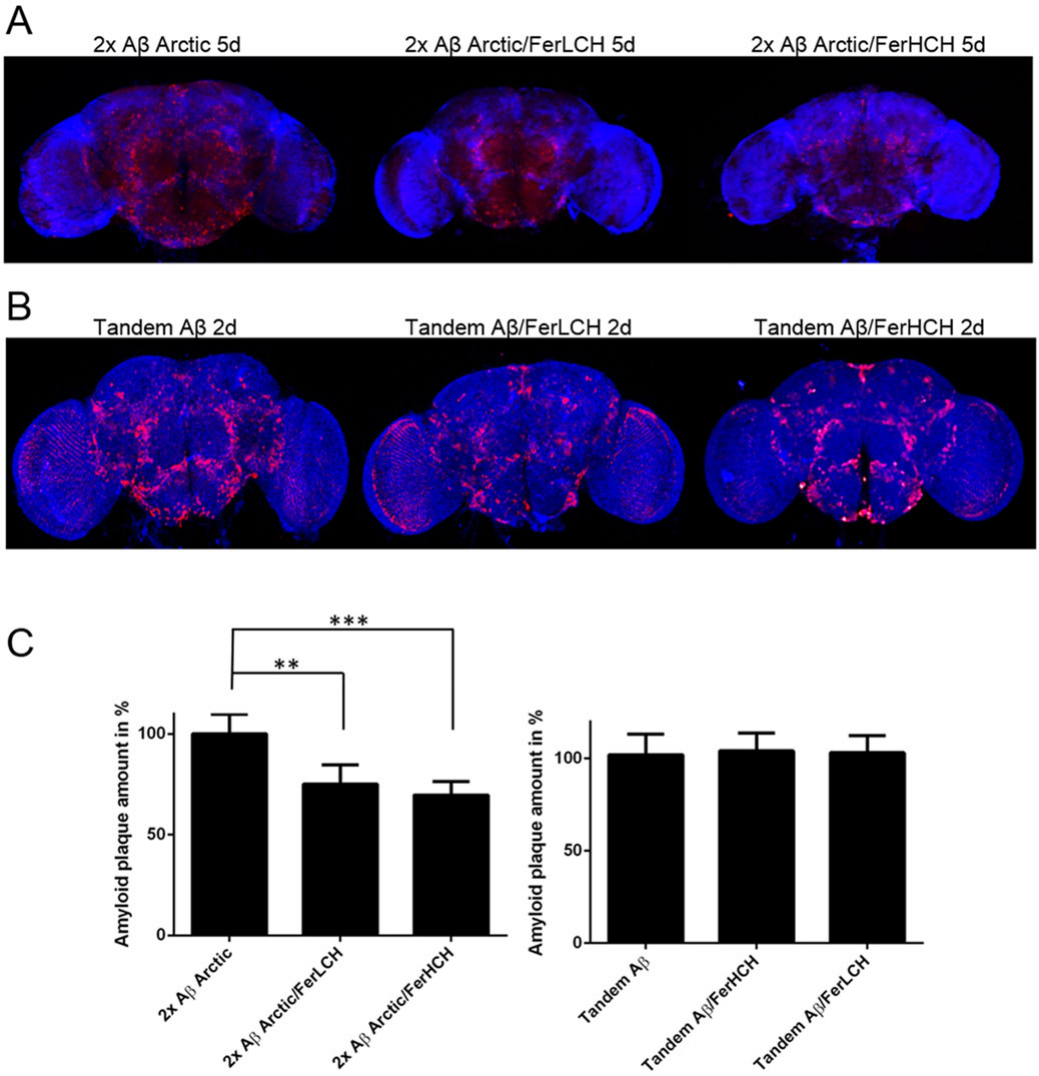
**Co-expression of ferritin subunits decreases the deposition of monomeric but not tandem Aβ.** (A) Fewer Aβ Arctic deposits are seen in *Drosophila* brains when ferritin is co-expressed. (B) Co-expression of either FerHCH or FerLCH did not alter the number of tandem Aβ_42_ brain deposits. Aβ is shown in red (6E10 anti-Aβ antibody) and nuclei (TOTO-3) in blue. Images are *z*-stack projections using identical microscope settings with a 10× objective. (C) Quantification of five representative fly brains revealed that ferritin reduces Aβ deposition when expressed as a monomer but not when expressed as the tandem construct. Statistical significance was determined by two-way ANOVA (**P<0.01, ***P<0.001). Error bars show s.d. ‘2×’ indicates two transgenes; otherwise, only one transgene is present.

To further investigate this relationship *in vivo*, we compared Aβ deposition in *Drosophila* brains expressing monomeric and tandem Aβ isoforms. Pan-neuronal expression of Aβ_42_ Arctic led to its accumulation and subsequent deposition in the fly brain (Fig. 5A). Detailed examination of the deposits by confocal microscopy revealed a mixture of larger, extracellular Aβ aggregates and smaller, ring-shaped inclusions that appeared to be intracellular (supplementary material Fig. S3). In contrast to monomeric Aβ_42_ Arctic, *Drosophila* expressing tandem Aβ exhibited more and larger deposits (Fig. 5B). The co-expression of ferritin subunits with monomeric Aβ_42_ resulted in reduced deposition, whereas no such effect was observed in the brains of flies expressing tandem Aβ (Fig. 5C). Importantly, the reduction in brain plaque numbers in *Drosophila* expressing monomeric Aβ was not caused by artefactual differences in gene expression, as determined by quantitative PCR for Aβ mRNA and western blotting (supplementary material Fig. S4).

### Position-dependent effects of His>Ala substitutions on Aβ toxicity in a non-oxidising environment

In this context, the N-terminal histidine residues at positions 6, 13 and 14 of the Aβ sequence are of particular interest because biophysical studies suggest that they are largely responsible for interactions between metal ions and Aβ (Minicozzi et al., 2008; Nair et al., 2010; Viles, 2012). To investigate the role of each histidine residue with regard to Aβ toxicity, we systematically substituted alanine residues for each of the histidines in Aβ and so created seven fly lines expressing, at equivalent levels and at the same genomic insertion site, all the possible variants of the peptide (supplementary material Fig. S5). Alanine was chosen as the substitute amino acid because it induced the least possible change in the predicted aggregation score according to the Tango algorithm (supplementary material Table S2) (Fernandez-Escamilla et al., 2004).

**Fig. 6.**
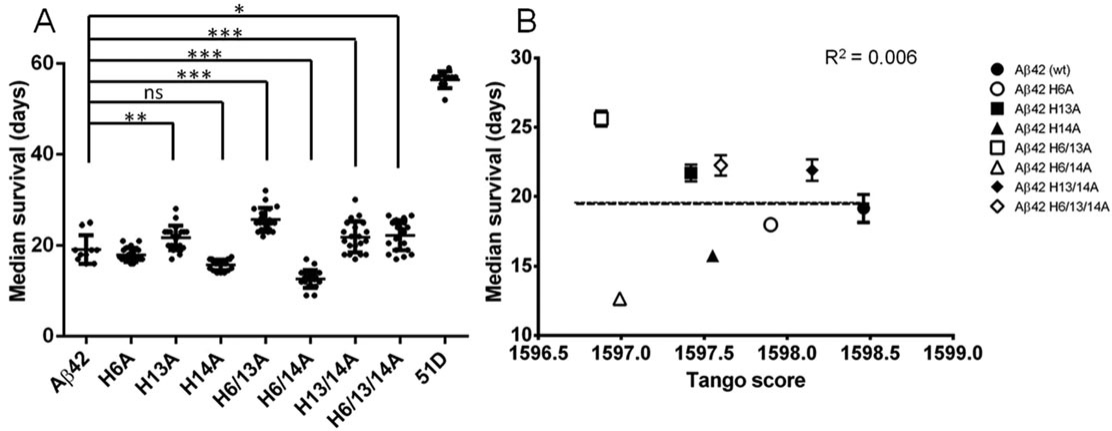
**Position-dependent effects of His>Ala substitutions on Aβ toxicity in a non-oxidising environment.** (A) His>Ala substitutions had strong position-dependent effects on fly longevity. The H13A substitution increased, and the H14A substitution decreased, the median survival. The H6A substitution did not have an effect alone; however, when combined with either H13A or H14A, H6A was able to potentiate the effect of the second mutation. (B) There was no correlation between the Tango aggregation propensity score for each peptide and the corresponding median survival of the flies expressing these variants. Flies were crossed at 25°C and maintained at 29°C after hatching. Differences in median survival were tested for significance using the Mann–Whiney test (ns, not significant; *P<0.5, **P<0.01, ***P<0.001). Error bars show s.d.

Eye-specific expression of wild-type and histidine variants of Aβ_42_ using GMR-gal4 did not cause a significant rough-eye phenotype (supplementary material Fig. S6). However, robust expression of the same Aβ peptides pan-neuronally using elav^c155^-gal4 resulted in moderate but significant reductions in longevity as compared with control flies. For example, the longest-lived flies (H6/13A, median survival=26 days) lived more than twice as long as the shortest-lived (H6/14A, median survival=12.5 days) (Fig. 6A). Moreover, the median survival of flies expressing the new His>Ala Aβ variants did not correlate with the small (∼0.2%) differences in the Tango aggregation propensity scores (Fig. 6B, R^2^=0.006). However, several observations can be made regarding the position-specific effects of the His>Ala substitutions. Considering the single mutants, it is apparent that H14A reduces longevity, H13A increases longevity and, by itself, H6A has no strong effect. In double mutants the presence of H6A enhances the effects of substitutions at positions 13 and 14. Thus, H6/13A is less toxic than H13A but H6/14A is more toxic than H14A. Where substitutions at both positions 13 and 14 occur in the same molecule, the effect of position 13 is dominant (supplementary material Table S2).

To determine whether any of these effects on longevity were accompanied by changes in the *in vivo* aggregation behaviour, we inspected the brains of *Drosophila* expressing the Aβ_42_ His>Ala variants pan-neuronally. Although there was no change in Aβ deposit numbers for most of the histidine exchange mutants, a striking difference was observed for the Aβ_42_ H6/14A construct (supplementary material Fig. S7G); in flies expressing this variant, quantification revealed more than twice as many Aβ deposits than in flies expressing the native Aβ_42_ isoform (supplementary material Fig. S7B).

### Position-dependent effects of His>Ala substitutions on the metal-dependent component of Aβ toxicity in a non-oxidising environment

**Fig. 7.**
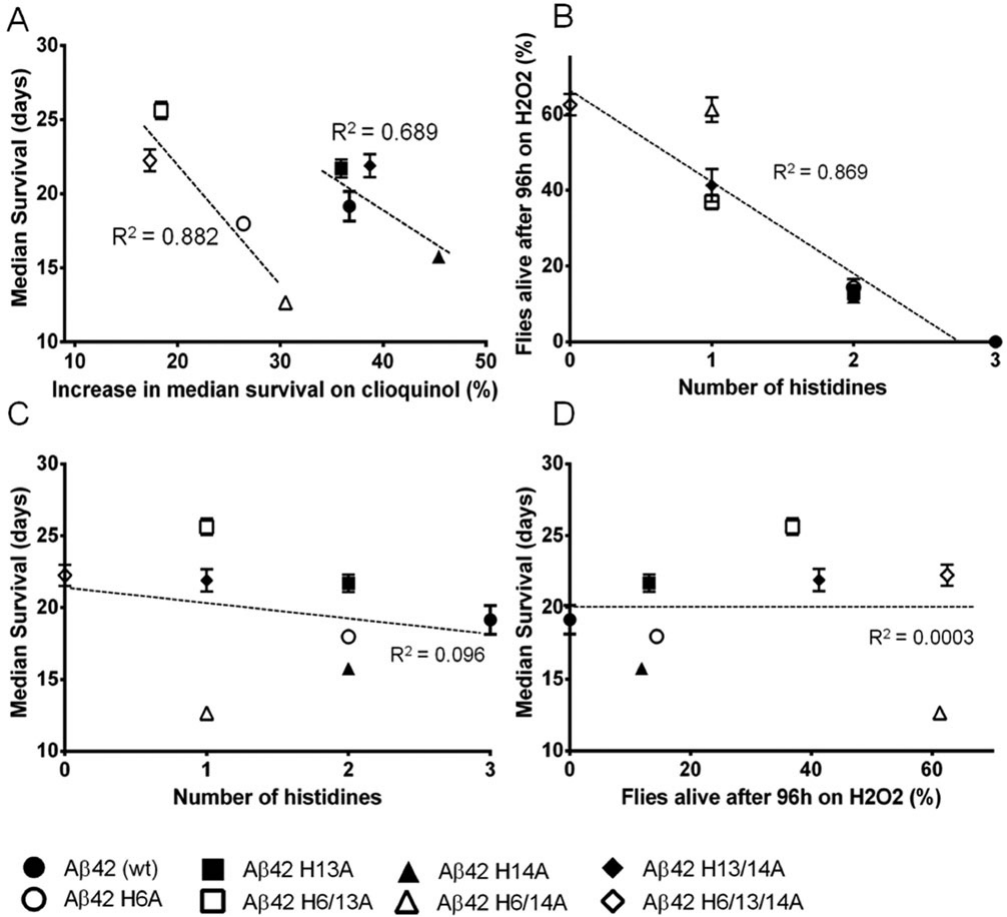
**Aβ_42_ His>Ala substitutions modify sensitivity to metal and oxidative toxicity.** (A) Flies expressing His>Ala variants combined with the H6A substitution were less sensitive than their His6 counterparts to the beneficial effect of metal chelation by clioquinol under non-oxidising conditions. Substitutions at positions 13 and 14 had position-specific effects that were conserved in the presence or absence of H6A. (B) There was a strong negative correlation between the total number of histidines in Aβ and the survival of the flies on hydrogen peroxide-supplemented food. Histidine substitution effects in an oxidising environment have position-independent effects on survival. (C) Under non-oxidising conditions, there was no relationship between the number of histidines in Aβ and its toxicity. (D) There was no correlation between the median survival of a given Aβ variant under non-oxidising conditions and the sensitivity of the corresponding fly to oxidative stress. Error bars show s.e.m. R^2^, correlation coefficient for the least squares linear regression.

We next assessed the effect of oral metal chelation on the toxicity of the various His>Ala Aβ variants. We calculated the percentage increase in the median survival of flies maintained on clioquinol-treated food, as compared with those fed normal food (supplementary material Table S2), and observed a weak overall correlation, such that flies with a short median survival responded better to clioquinol treatment (Fig. 7A, R^2^=0.2, linear fit for all points not shown). However, the data could be resolved into two much stronger subcorrelations (R^2^=0.689 and R^2^=0.882, linear fits shown). The approximately parallel linear fit lines (Fig. 7A) indicate that, for any given effect of substitutions at positions 13 and 14, the addition of the His>Ala exchange at position 6 caused a 15-20% reduction in response to the life-prolonging effects of clioquinol.

### Position-independent effects of His>Ala substitutions on Aβ toxicity in an oxidising environment

Finally, the effects of oxidative stress were estimated from the survival rate of young flies that were kept on food supplemented with hydrogen peroxide. All of the His>Ala variants resulted in greater resistance to oxidative stress than flies expressing the native Aβ_42_ peptide (supplementary material Fig. S8). When we tabulated the percentage of flies surviving at 96 h, we observed a robust correlation with the number of histidines in each Aβ isoform (irrespective of their position in the peptide). Flies with more histidines showed greater sensitivity to oxidative stress (Fig. 7B). By contrast, the number of histidines did not correlate with the median survival of flies in a non-oxidising environment (Fig. 7C).

We found no cross-correlation between the position-dependent and -independent phenotypes. For example, the median survival under non-oxidising conditions did not correlate with the survival of flies in an oxidising environment (Fig. 7D, R^2^=0.0003), whether or not we grouped the data according to the presence of the H6A substitution.

## DISCUSSION

A number of neurodegenerative disorders are characterised by the abnormal metabolism of metals, such as copper and iron; however, the relevance of this observation to pathological mechanisms remains unclear. Consequently, we have used several models of protein aggregation disorders to investigate the role of metals. In our previous work using a *Drosophila* model of Aβ toxicity we found that the iron-binding protein ferritin and other iron-chelators are powerful suppressors of both Aβ toxicity and the associated markers of oxidative damage (Liu et al., 2011; Rival et al., 2009). Consequently, here we have asked whether ferritin expression also rescues disease-related phenotypes in other *Drosophila* models of common neurodegenerative disorders. Specifically, we have studied flies expressing tau, the Q48 peptide and TDP43, in addition to various isoforms of Aβ.

The inclusion of these particular polypeptides in the study is justified by varying degrees of evidence linking their pathogenicity to interactions with metal cofactors. Considering tau, its hyperphosphorylation and deposition as neurofibrillary tangles (Spillantini and Goedert, 2013) are characteristic of AD and other tauopathies. Some investigators have highlighted the role of metal binding (García de Ancos et al., 1993; Huang et al., 1999a; Ledesma et al., 1995; Ma et al., 2006, 2005) in generating tau deposits (Zhou et al., 2007) and the consequent neuronal microtubule dysfunction (Craddock et al., 2012). It is thought that this process is induced by the ability of Fe^3+^ to bind to phosphorylated tau, causing it to aggregate (Bader et al., 2011; Nübling et al., 2012; Yamamoto et al., 2002). Furthermore, tau, like Aβ, may form complexes with iron and copper, resulting in the generation of reactive oxygen species (Sayre et al., 2000; Su et al., 2007), in turn favouring tau phosphorylation (Chan and Shea, 2006; Egaña et al., 2003; Lovell et al., 2004).

Similarly, in HD there is evidence for abnormal metal metabolism (Dexter et al., 1991, 1992), with clinical MRI scans showing iron accumulation in susceptible brain regions (Bartzokis et al., 1999, 2007). Iron levels tend to be higher in advanced disease and in patients with longer polyQ repeat lengths (Rosas et al., 2012). In mouse (Fox et al., 2007) and fly (Xiao et al., 2013) models of HD there is good evidence that both copper and iron metabolism are disturbed and that chelator therapy may be beneficial (Nguyen et al., 2005). *In vitro*, metals promote oxidative damage, with a truncated huntingtin polypeptide interacting with both Cu^2+^ and Fe^3+^ (Fox et al., 2007, 2011).

Finally, familial amyotrophic lateral sclerosis (ALS) may result from mutations in the genes for TDP43 (Sreedharan et al., 2008), FUS (Kwiatkowski et al., 2009; Vance et al., 2009), C9ORF72 (DeJesus-Hernandez et al., 2011; Renton et al., 2011) and, classically, SOD1 (Rosen et al., 1993). Although there is considerable evidence that copper plays a role in the pathogenicity of SOD1 variants (Barnham and Bush, 2014), the role of metals in ALS as a whole is less clear. For example, mice expressing disease-linked variants of TDP43 show abnormal metabolism of a number of metals, although iron appears to be normal (Dang et al., 2014).

Considering this evidence that iron is more or less important in the pathological actions of a series of aggregation-prone polypeptides, it is remarkable that, in this study, iron chelation by ferritin was beneficial only for flies expressing Aβ. Our finding that ferritin offered Aβ-specific rescue from both longevity and eye phenotypes indicates that an important component of Aβ toxicity *in vivo* is not generic. Thus, the overall toxicity of an aggregating polypeptide seems to be composed of a core generic component, which is likely to be related to the presence of oligomeric aggregates. To this core are added peptide-specific effects that are modulated by environmental cofactors – in this case the presence of iron or other metals. One way in which metals could enhance Aβ toxicity is by stabilising particular conformers in the aggregation pathway. The formation of an initial dimer is likely to be a fundamental step and we were able to investigate this *in vivo* by expressing either the normal monomeric peptide or a ‘pre-dimerised’ tandem Aβ peptide. We have previously shown that both tandem Aβ_42_ and tandem Aβ_40_ aggregate rapidly *in vivo*; however, of these, only the 42 amino acid isoform is able to generate stable oligomeric aggregates and exhibit pronounced toxicity (Speretta et al., 2012). Here, we present tentative evidence *in vitro* that amyloid generation by partially purified, recombinant Aβ and by tandem Aβ responds differently to the addition of iron (Fig. 3). Specifically, the addition of iron to a preparation of partially purified tandem Aβ does not cause the slowing of ThT amyloid signal that was observed with the monomeric peptide preparation (Liu et al., 2011). Concordant with these *in vitro* results, we find that the toxicity associated with tandem Aβ in the fly is not suppressed by co-expression of ferritin. Furthermore, ferritin had no effect on the number of tandem Aβ deposits in the brain, whereas flies expressing monomeric Aβ consistently had up to 25% fewer deposits in the presence of ferritin (Figs 4 and 5). Importantly, this reduced plaque load was not due to decreased Aβ transgene expression or peptide production. In summary, these results suggest that, both *in vitro* and *in vivo*, iron is interacting with Aβ to accelerate a step that is redundant in the tandem peptide; this step is likely to be the formation of dimeric aggregates. These results in a *Drosophila* model are reminiscent of the reduction in plaque load in tg2576 mice treated with the broad-spectrum metal chelator clioquinol (Cherny et al., 2001) and support the relevance of this study for mammalian systems.

After evaluating the specificity of iron for Aβ-induced toxicity and its effect on aggregation, we focussed our efforts on studying potential interactions between Aβ and iron. Such interactions are thought be mediated by the three N-terminal histidine residues at positions 6, 13 and 14 (Nakamura et al., 2007). We systematically assessed how each of the seven possible combinations of His>Ala substitutions modifies the elaboration of three key disease-linked phenotypes, namely: (1) survival in an oxidising environment; (2) survival in a non-oxidising environment; and (3) the proportion of Aβ toxicity that is metal dependent.

To assess the response of the flies to oxidative stress we measured survival after oral challenge with hydrogen peroxide. Whereas control flies are robust when treated in this way, there is a marked reduction in survival after 78 and 96 h when Aβ is expressed (Fig. 7B; supplementary material Fig. S8). Remarkably, only the number of histidines in a particular Aβ variant determined the susceptibility to oxidative stress. This histidine dose dependency and position independence makes it unlikely that the mechanism involves specific changes in peptide conformation or overall aggregation propensity; indeed, the hydrogen peroxide feeding experiments are performed in 6-day-old flies in which there is no detectable peptide aggregation. Moreover, the predicted aggregation propensities of the various peptide isoforms are essentially identical (supplementary material Table S2). A possible molecular scale interpretation of these results is that the formation of redox-active metal-peptide complexes is rather flexible and peptide promiscuous. In this model, histidines from distinct peptides could co-ordinate to a shared metal ion and generate a redox-active complex. An alternative explanation might be that there is an oxidative reaction in the presence of Aβ that uses histidines as a substrate, thereby generating toxic oxygen species (Cheng et al., 1992). In either case, these results predict that reducing total brain Aβ levels might be most effective in reducing the oxidative damage in AD.

By contrast, the remaining two disease-linked phenotypes are sensitive to the positions of each of the His>Ala substitutions. In a non-oxidising environment the survival of flies expressing the various peptides is determined by position-dependent factors. Some *in vitro* studies have shown that the histidines at positions 13 and 14 interact similarly with copper in models of amyloid fibrils (Parthasarathy et al., 2011), whereas others suggest that the H14A substitution in synthetic Aβ_42_ reduces toxicity, possibly by preventing the interaction of the peptide with cell membranes (Smith et al., 2010). The longevity effects observed in our experiments, although relatively modest, were nevertheless both robust and significant, and presented us with a contrasting observation. Specifically, we found that H14A enhances toxicity, as evidenced by a reduction in longevity, whereas the H13A substitution has the opposite effect. Interestingly, the H6A substitution has a modulating role in our fly model; in particular, H6A acts to amplify the longevity effects of substitutions at positions 13 and 14. Accordingly, H14A reduces the median survival and H6/14A has the shortest lifespan of all the fly lines. By contrast, *Drosophila* expressing the H13A variant live longer than wild-type flies and H6/13A live the longest of all the peptide-expressing lines. H6 also modulates the accumulation of peptide deposits in the brain, with H6/14A showing remarkably high levels of Aβ deposition, higher than H14A alone. The use of the φC31 system to target all of the Aβ transgenes to the same 51D genomic insertion site in the same acceptor fly line makes it highly unlikely that the phenotypic differences observed here are due to artefactual fluctuations in transgene expression levels. Furthermore, the genetic background of the experimental flies should be essentially identical. It should also be noted that the density of Aβ deposits was comparable between lines despite highly significant differences in median survival (Fig. 6; supplementary material Fig. S7).

The modulating role of H6 is seen most clearly in the third phenotype, where we measured the degree to which the metal chelator clioquinol prolongs the longevity of the various fly lines. When we calculate the percentage increase in median survival upon treatment with clioquinol, it is apparent that the histidines at positions 13 and 14 each have their position-dependent effects on the clioquinol response. However, there is an overwhelming additive effect of histidine at position 6, such that each corresponding H6A variant shows a 25-30% reduction in clioquinol responsiveness (Fig. 7A). The implication of these results is that much of the metal-mediated toxicity, in a non-oxidising environment, is dependent on H6A and that, thereafter, there are smaller position-dependent effects of residues 13 and 14. A possible mechanistic interpretation is that metal interactions with H6 initiate metal-mediated Aβ dimerisation and, thereafter, histidines 13 and 14 determine the particular conformations of downstream aggregates and their toxicity (supplementary material Fig. S9). These position-dependent properties of individual histidine residues make it likely that particular peptide conformations are mediating the combined Aβ-iron effects; such structures might be amenable as therapeutic targets.

In summary, our systematic study of the effects of metals and their specific interactions with Aβ *in vivo* indicates that dimerisation of the peptide, perhaps mediated by H6, is an early step in generating toxicity. The subsequent toxic consequences of Aβ aggregation are then largely determined by the histidines at positions 13 and 14. In a strongly oxidising environment the importance of peptide aggregation is complemented, if not overwhelmed, by the histidine-dependent oxidative damage mediated by Aβ.

## MATERIALS AND METHODS

### Materials

All materials were sourced from Sigma-Aldrich unless otherwise stated.

### Fly stocks

*Drosophila* FerHCH and FerLCH stocks have been described previously (Rival et al., 2009). The UAS-Arctic Aβ_42_, UAS-Aβ_42_ and UAS-Aβ_40_ transgenic flies that were used for all survival assays were created by site-directed insertion at the 51D location on the second chromosome using the φC31 integrase system as previously described (Bischof et al., 2007; Kruppa et al., 2013). The ‘pre-dimerised’ tandem Aβ_42_ isoform (with a 12 amino acid linker peptide) has been described previously (Speretta et al., 2012). Previously described *Drosophila* lines were used for Aβ (AlzArc1 and AlzArc2 constructs) (Crowther et al., 2005), tau (Wittmann et al., 2001), TDP43 (Gregory et al., 2012) and Q48 (Steffan et al., 2001) expression.

### Longevity assays

Longevity assays were performed as described previously (Crowther et al., 2005). All experiments were carried out with mated female *Drosophila* in which at least 90 flies, sorted into groups of ten, were tested for each respective genotype. Unless otherwise indicated, animals were cultured at 29°C throughout. Differences in survival were visualised using Kaplan–Meier survival plots. Statistical analysis of median survival assumed that each tube of ten flies provided an estimate of the population median survival. The Mann–Whitney test was used to assess the statistical significance of differences in the estimated median survival of each genotype. At least nine tubes of ten flies were assessed per genotype.

### Clioquinol feeding experiments

Oral metal chelation was undertaken by supplementing standard fly medium [1% agar, 8% glucose, 8% maize, 1.5% yeast, 0.25% methylparaben (all w/v)] with the broad-spectrum metal chelator clioquinol (200 µM). Clioquinol was dissolved in 80% (v/v) DMSO before addition to 1 litre of freshly prepared liquid fly food and mixed before being dispensed into individual fly culture tubes.

### Generation of Aβ His>Ala exchange constructs

Aβ_42_ histidine-to-alanine (His>Ala) substitutions were created by site-directed mutagenesis (QuikChange kit, Agilent), according to the manufacturer's protocol. The reaction was carried out using mutagenic primers (supplementary material Table S1) using pUC19 vector (GenBank L09137) that contained the insect codon-optimised, native Aβ_42_ sequence. Successful mutagenesis was confirmed by DNA sequencing (Source Bioscience) of the insert. The Aβ_42_ His>Ala constructs were subcloned into a pUAST-AttB vector (GenBank EF362409) and transgenic *Drosophila* lines were created by site-directed genomic insertion using the φC31 system at the 51D location on the second chromosome as described previously (Bischof et al., 2007; Kruppa et al., 2013). Plasmids carrying multiple site-directed mutations were made by sequential rounds of mutagenesis.

### Hydrogen peroxide feeding

Two-day-old mated female flies were sorted into vials in groups of 20 and cultured at 25°C throughout the experiment. The control population was maintained on 2% (w/v) agar that was dissolved in 5% (w/v) sucrose in water. In the test population, the food was supplemented with 10% (v/v) hydrogen peroxide. Experiments were performed with at least 160 flies per genotype and the survival characteristics were recorded twice per day. Differences in survival between genotypes were analysed for statistical significance by two-way ANOVA.

### Brain dissections

Brain dissections were performed as described previously (Costa et al., 2011). Aβ plaques in *Drosophila* brains were quantified by using ImageJ (NIH). Brain plaque quantification was only undertaken on images that were acquired under identical conditions and on the same day. At least four different brain samples were quantified for each genotype. Differences in plaque counts were tested for significance using two-way ANOVA. To probe whether Aβ deposits were intracellular or extracellular, *Drosophila* brains were stained with TOTO-3 (Life Technologies) for nuclear staining and with phalloidin (Life Technologies) to label intracellular actin. The presence of Aβ deposits was detected with the 6E10 monoclonal antibody (Covance, SIG-39300, 1:100 dilution). Aβ deposits were assessed across consecutive 1 µm confocal slices to determine whether they cross or remain within cellular boundaries.

### Quantitative PCR

Quantitative PCR was performed as previously described (Kruppa et al., 2013).

### Western blot

Western blots on fly head homogenates were carried out as previously described (Speretta et al., 2012).

### Aβ expression and purification in bacterial cells

*E. coli* cultures [Tuner(DE3), Merck Millipore] carrying expression plasmids for Aβ_42_ or tandem Aβ_42_ were cultured at 37°C on a shaker in 2 litres of lysogeny broth (LB) supplemented with carbenicillin. At an OD_600 nm_ of 0.6, 1 mM isopropyl β-D-1-thiogalactopyranoside (IPTG) was added to the cultures to induce vector expression. Four hours after induction the bacterial culture was harvested by centrifugation at 10,000 ***g*** and the supernatant discarded. The pellet was resuspended in 10 ml lysis buffer [1 mM EDTA, 100 mM NaCl, 1% (v/v) Triton X-100, 50 mM Tris-HCl, pH 9]. The resulting bacterial lysate was sonicated for 3 min and then centrifuged at 18,000 ***g*** for 30 min and the supernatant discarded. The pellet was resuspended in lysis buffer, sonicated and centrifuged twice more, each time retaining the washed pellet. Finally, the pellet was resuspended in 1 ml 100% DMSO and stored at −20°C.

### Thioflavin T assays with bacterial Aβ extracts

Aggregation assays using partially purified Aβ extracts were carried out on a FLUOstar Optima plate reader (BMG Labtech) as described previously (Liu et al., 2011). The total protein concentration in the bacterial extracts was quantified by the DC assay (Bio-Rad). The final protein concentration was 2 µM for the Aβ_42_ extracts and 1 µM for the tandem Aβ_42_ extracts. In all assays, FeCl_3_ in modified Krebs-Henseleit buffer (123 mM NaCl, 4.8 mM KCl, 1.2 mM MgSO_4_, 1.4 mM CaCl_2_, 11 mM glucose, 100 mM PIPES, pH 7.4) was used to study the effect of iron on Aβ aggregation. All conditions were tested with at least three replicate samples and repeated twice.

## Supplementary Material

Supplementary Material
