# Supplementary material for: Iron is a specific cofactor for distinct oxidation- and aggregation-dependent Aβ toxicity mechanisms in a Drosophila model
Source: Dis Model Mech. 2015 Jul 1;8(7):657–67. doi: 10.1242/dmm.019042 (PMC4486857; doi:10.1242/dmm.019042)
Supplement: Supplementary Material [file supp_8_7_657__index.html]

Supplementary Material 

# Iron is a specific cofactor for distinct oxidation- and aggregation-dependent Aβ toxicity mechanisms

## DMM019042 Supplementary Material

- Supplementary Material
