# Supplementary material for: Iron is a specific cofactor for distinct oxidation- and aggregation-dependent Aβ toxicity mechanisms in a Drosophila model
Source: Dis Model Mech. 2015 Jul 1;8(7):657–67. doi: 10.1242/dmm.019042 (PMC4486857; doi:10.1242/dmm.019042)
Supplement: Supplementary Material [file supp_019042_DMM019042supp.pdf]

Table S1: Sequences of primers used in site-directed mutagenesis

Construct	Primer sequence	Orientation
H6A	5' AGGATGCGGAATTTCGCGCGGACAGCGGCTACGAAGT 3'	Forward
H6A	5' ACTTCGTAGCCGCTGTCCGCGCGAAATTCCGCATCCT 3'	Reverse
H13A	5' ACAGCGGCTACGAAGTGGCGCATCAAAAATTGGTGTT 3'	Forward
H13A	5' AACACCAATTTTTGATGCGCCACTTCGTAGCCGCTGT 3'	Reverse
H14A	5' CAGCGGCTACGAAGTGCATGCGCAAAAATTGGTGTTTTTTG 3'	Forward
H14A	5' CAAAAAACACCAATTTTTGCGCATGCACTTCGTAGCCGCTG 3'	Reverse
H13/14A	5' CAGCGGCTACGAAGTGGCGGCGCAAAAATTGGTGTTTT 3'	Forward
H13/14A	5' AAAACACCAATTTTTGCGCCGCCACTTCGTAGCCGCTG 3'	Reverse
H6/13/14A	5' GCGGAATTTCGCGCGGACAGCGGCTACGAAGTGGCGGCGCAAAAATTGGTG 3'	Forward
H6/13/14A	5' CACCAATTTTTGCGCCGCCACTTCGTAGCCGCTGTCCGCGCGAAATTCCGC 3'	Reverse

Table S2: Characteristics of Aβ$_{42}$ His>Ala constructs

Aβ variant	Tango score	Flies alive on H_2O_2 after 96 hr (%)	Median survival (days)	Number of histidines in Aβ	Increase in median survival on clioquinol (%)
Aβ$_{42}$ (wt)	1598.46	0	18	3	36.7
H6A	1597.9	14.4	18	2	26.4
H13A	1597.42	13.1	21.5	2	35.9
H14A	1597.55	11.9	15.5	2	45.4
H6/13A	1596.88	36.9	26	1	18.4
H6/14A	1596.99	61.3	12.5	1	30.5
H13/14A	1598.15	41.3	21.5	1	38.7
H6/13/14A	1597.6	62.5	22	0	17.3
51D control	N/A	58.4	70	N/A	-2.8

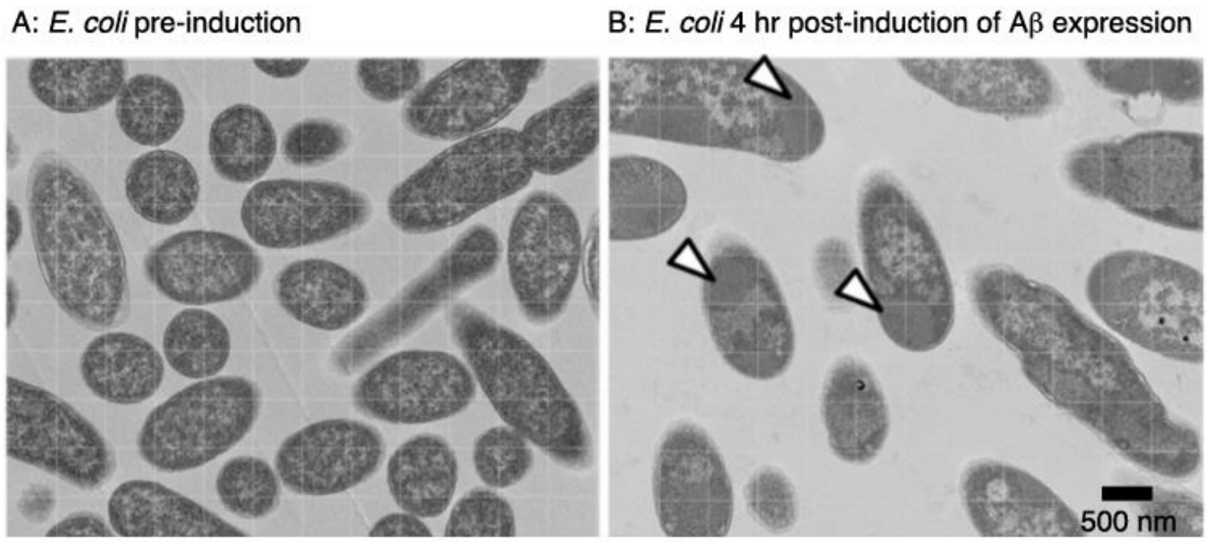

Figure S1: Bacterial cultures deposit expressed $A\beta_{42}$ in inclusion bodies

E. coli expressing $A\beta_{42}$ were cultured to an $OD_{600\ nm}$ of 0.2 before induction with 1 mM IPTG for 4 hrs. Subsequently, culture samples were examined by transmission electron microscopy. To minimise bias in collecting the samples, the electron micrographs were obtained by taking images of each corner of the grid subdivisions. In total, 10-15 images of bacteria expressing each Aβ isoform were taken at each time point. The electron microscopic appearance of transfected *E. coli* before induction (A) was normal, whereas after 4 hr induction of Aβ expression intracellular inclusion bodies were visible (B).

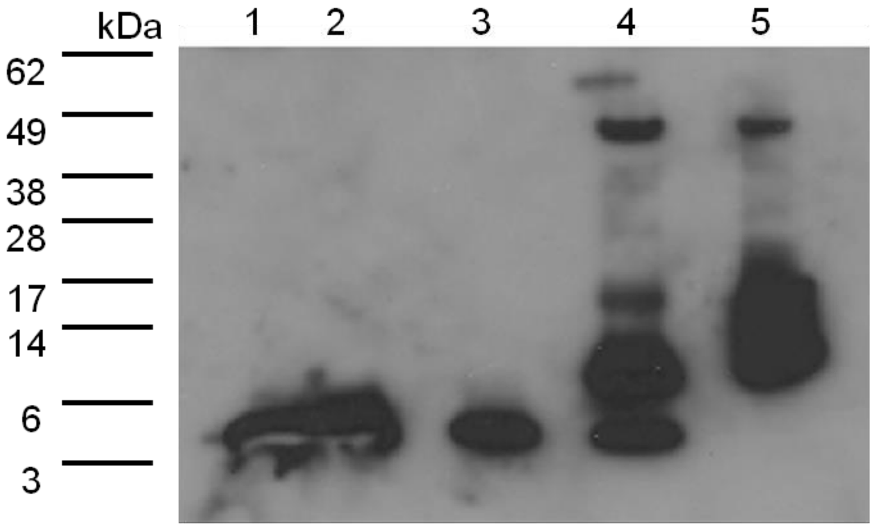

Figure S2: Bacterial extracts expressing different Aβ isoforms

The figure illustrates a western blot of a 2% SDS soluble Aβ fraction which was purified from *E. coli* inclusion body samples. Legend: 1= $A\beta_{40}$, 2= $A\beta_{42}$, 3= $A\beta_{42}$ Arctic, 4 = tandem $A\beta_{40}$, 5= tandem $A\beta_{42}$. As shown in lanes 1-3, bacterial cultures expressing monomeric Aβ produced an Aβ band in the 5-7 kDa region. Tandem Aβ species were detected at the 14 kDa region (lanes 4 and 5). The western blot shows that Aβ produced by bacteria was either monomeric or dimeric and therefore suitable for further studies in ThT assays. The primary anti-Aβ monoclonal antibody used for detection was 6E10.

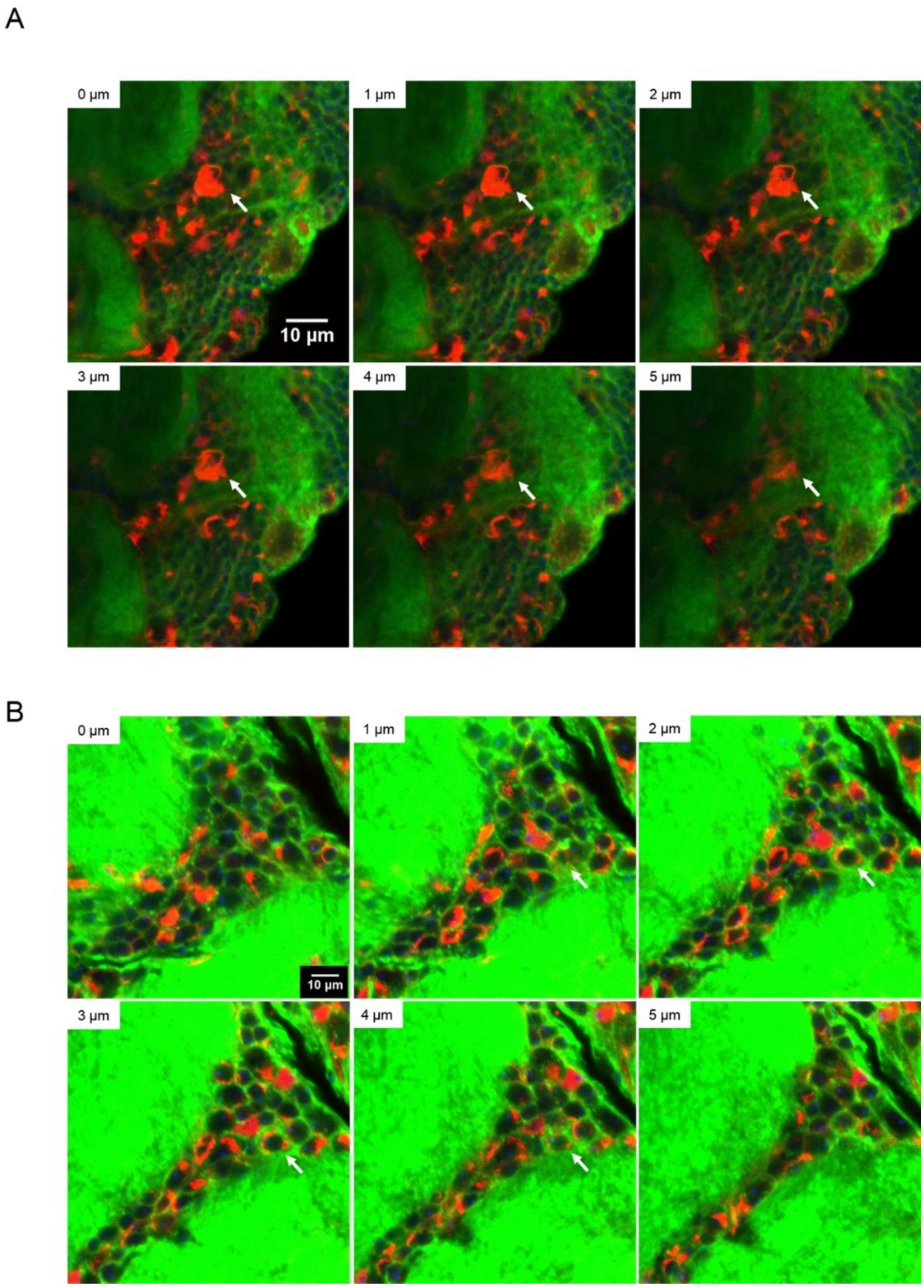

Figure S3: Expression of Aβ leads to the deposition of intra- and extra-cellular plaques

The figure illustrates examples of intra- and extra-cellular Aβ deposits. Both panels show single images of a *z*-stack with a slice size of 1 µm. The images are arranged from top left to bottom right in order of the *z* projection. A deposit of interest is indicated by a white arrow next to it. Panel A shows the example of an extracellular and panel B the example of an intracellular Aβ plaque. Aβ deposits are visualized in red nuclear TOTO-3 staining in blue and phalloidin in green.

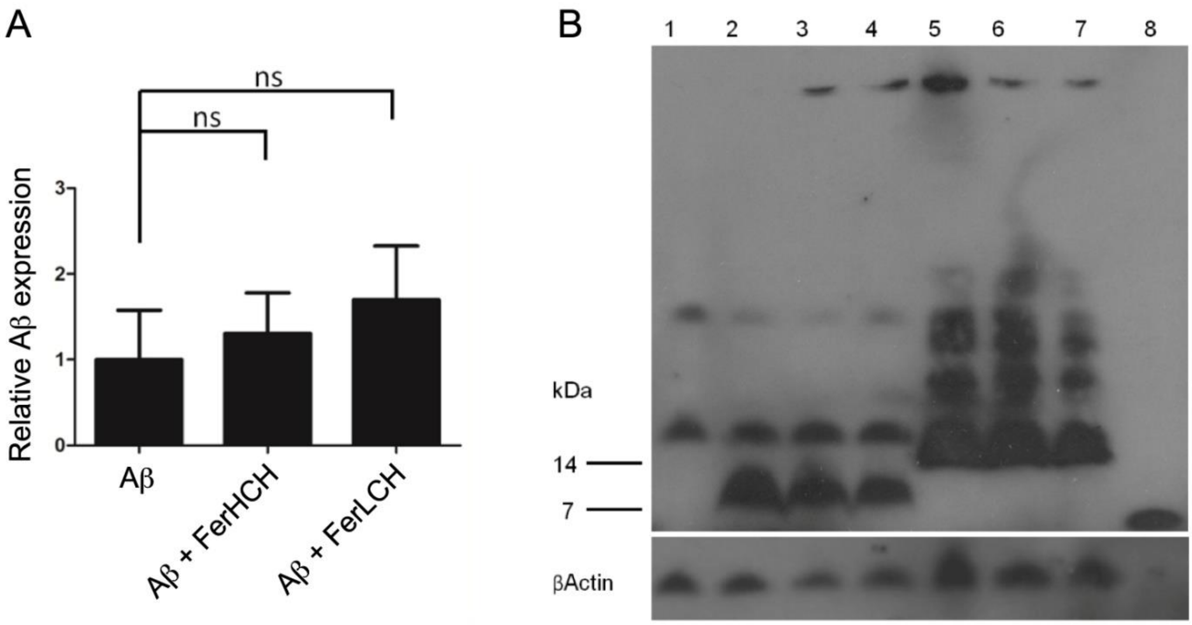

Figure S4: Ferritin co-expression does not change the levels of Aβ gene expression or production

Panel A: Co-expression of ferritin subunits did not change the expression levels of $A\beta_{42}$ Arctic. Quantitative PCR for Aβ mRNA was performed on extracts of heads from 12 day old flies that were aged at 29°C. *RP-49* was used as a background control. The experiment was performed twice with triplicate samples for each genotype. Panel B: Ferritin co-expression did not alter the levels of monomeric and tandem Aβ in *Drosophila* head homogenates. The western blot for 2% SDS-soluble Aβ was carried out with 20 day old flies which were aged at 29°C. Legend: 1 = control fly extract, 2 = 2x $A\beta_{42}$ Arctic, 3= 2x $A\beta_{42}$ Arctic/FerHCH, 4= 2x $A\beta_{42}$ Arctic/FerLCH, 5= tandem $A\beta_{42}$, 6= tandem $A\beta_{42}$/FerHCH, 7= tandem $A\beta_{42}$/FerLCH, 8= positive control (synthetic $A\beta_{42}$). The $A\beta_{42}$ Arctic band is detected in lanes 2-4 just above the 7 kDa marker, tandem $A\beta_{42}$ runs as a 14 kDa dimer (lanes 5-7). The lower panel shows the β-actin control for each lane. The primary anti-Aβ monoclonal antibody was 6E10. “2x” in the panel titles indicates two transgenes; otherwise only one transgene is present.

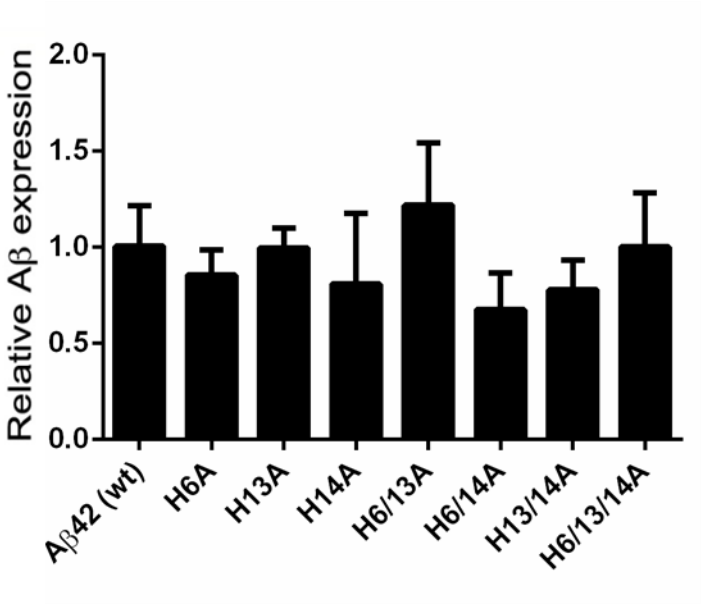

Figure S5: *Drosophila* expressing His>Ala exchange variants and the native Aβ_{42} show comparable levels of gene expression

Quantitative PCR for Aβ mRNA indicates that flies are expressing the different Aβ variants at equivalent levels. The PCR was performed with 15 day old flies that were aged at 29°C. Expression levels of the house keeping gene *RP-49* were used as a background control. Five replicate samples were tested for each genotype and the experiment was carried out twice.

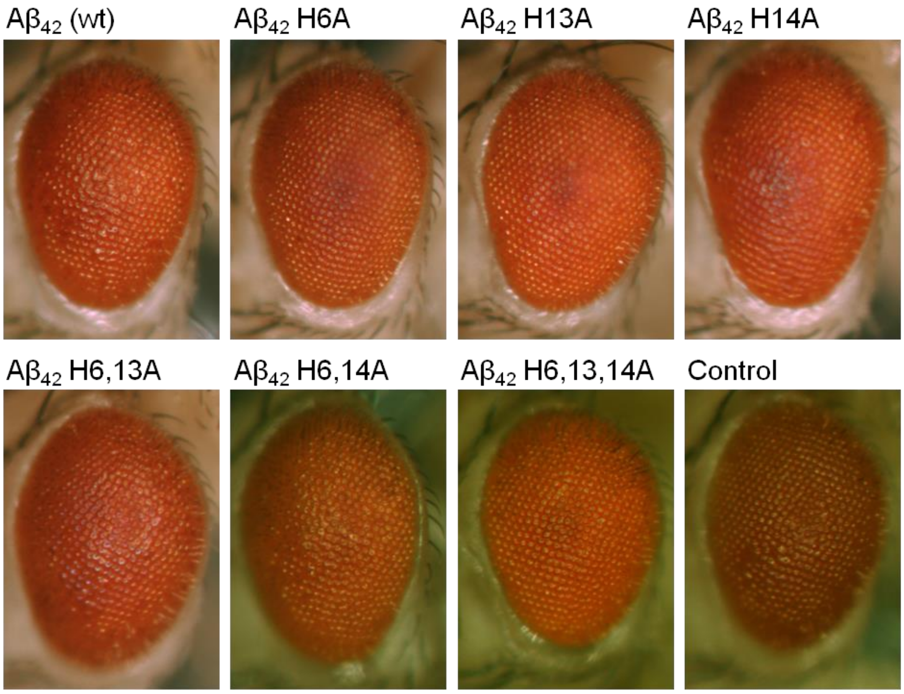

Figure S6: Eye-specific expression of wild type, and the His variants of, $A\beta_{42}$ using GMR-gal4 did not cause a significant rough eye phenotype.

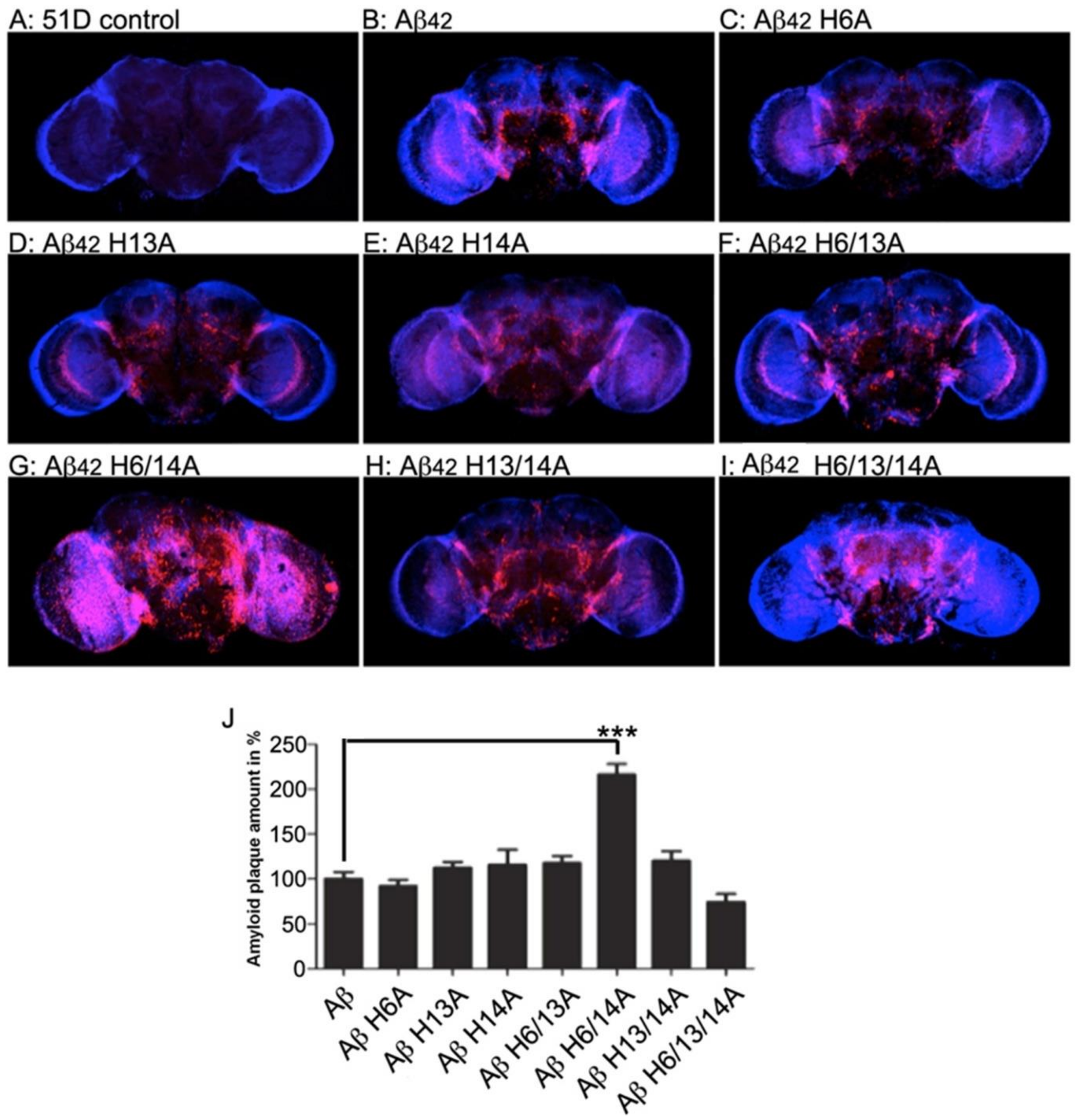

Figure S7: His>Ala substitutions modify Aβ_{42} deposition in *Drosophila* brains

Panels A-I show confocal brain images of flies expressing different Aβ_{42} His>Ala variants pan-neuronally. Aβ plaques are shown in red (4G8 anti Aβ antibody) and the background staining (DNA staining with TOTO-3) in blue. At least five fly brains per condition were imaged and their Aβ plaque numbers quantified. All flies were aged to 10 days at 29 °C prior to dissection. Panel J: Differences in Aβ plaque numbers were examined by two-way ANOVA (*** $P < 0.001$). Error bars show the standard deviation.

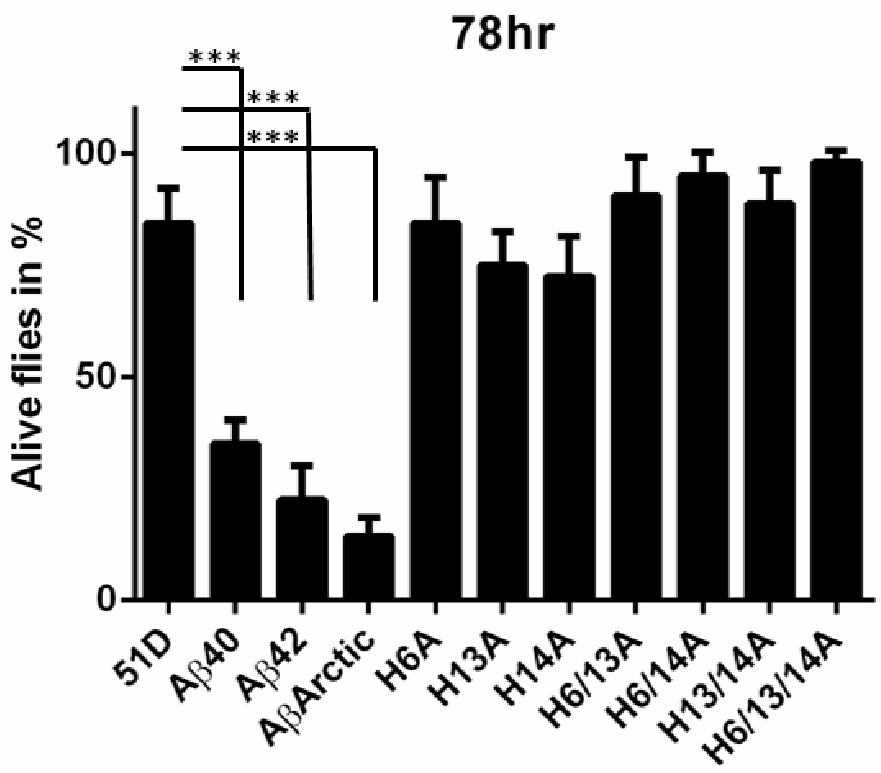

Figure S8: *Drosophila* expressing His>Ala variants are more resistant to oxidative stress

Flies expressing any of the $A\beta_{42}$ His>Ala variants were more resistant to oxidative stress than those expressing either $A\beta_{40}$, $A\beta_{42}$ or $A\beta_{42}$ Arctic. The assay was performed with 160 flies per genotype. There were no deaths in the control population throughout the experiment (data not shown). Differences in survival proportions were examined by two-way ANOVA (*** $P < 0.001$). Error bars show the standard deviation.

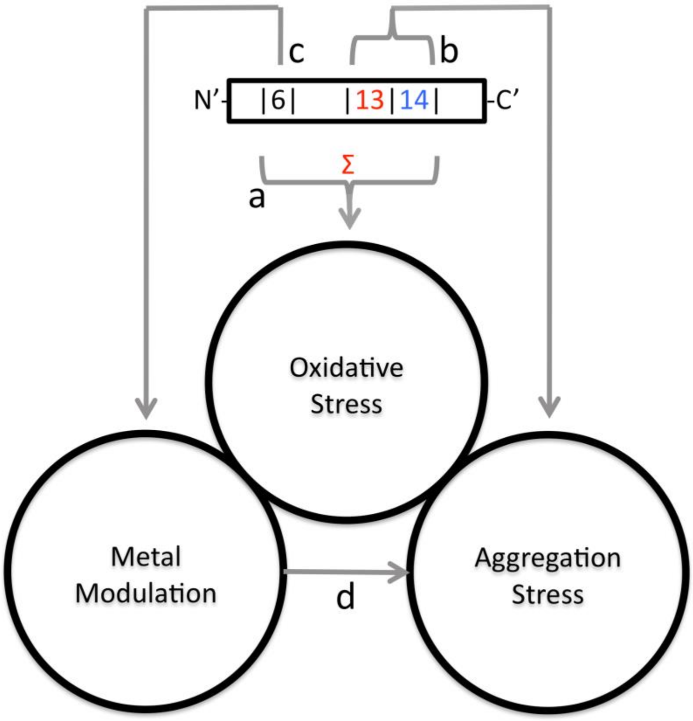

Figure S9: The toxicity of Aβ in vivo has three components that are differentially mediated by the N-terminal histidines at position 6, 13 and 14.

The lethality of an oxidative challenge is mediated in a position-independent manner (a) and correlates with the total number of histidines (Σ) present in a particular Aβ variant (N'…C'). The *in vivo* toxicity of the Aβ variants is specifically modified by H13 and H14 (b); specifically H13 favours decreased longevity whereas H14 favours increased longevity. These different effects of H13 and H14 are both enhanced upon addition of the H6A substitution (c & d).
